# Erythrocytes and cardiovascular complications

**DOI:** 10.18632/aging.101688

**Published:** 2018-11-28

**Authors:** Zhichao Zhou, Jiangning Yang, John Pernow

**Affiliations:** 1 Division of Cardiology, Department of Medicine, Karolinska University Hospital, Karolinska Institutet, Stockholm 17176, Sweden

**Keywords:** erythrocyte, cardiovascular complications, arginase, nitric oxide, ATP, reactive oxygen species

Erythrocytes play a fundamental role in cardiovascular homeostasis, contributing to vascular function and integrity. Erythrocytes undergo functional alterations including reduced nitric oxide (NO)-like bioactivity and/or enhanced oxidative stress in various diseases including myocardial infraction, pulmonary hypertension, diabetes, and hemoglobinopathies [1]. Such altered function of erythrocytes may subsequently affect cardiovascular function thereby accounting for cardiovascular complications.

Impaired NO bioavailability occurs early and contributes to progression and prognosis of cardiovascular complications. NO is produced from L-arginine by endothelial NO synthase (eNOS) which competes with arginase for the common substrate L-arginine [2]. In contrast to the early concept that erythrocytes serve as NO “sink”, functional eNOS was found to be present in human and mouse erythrocytes [1]. Of particular importance, we provided evidence that erythrocytes release NO-like bioactivity for cardiac function regulated by erythrocyte arginase [3]. We demonstrated that arginase inhibition in erythrocytes significantly improved post-ischemic functional recovery in isolated rat hearts. The cardioprotective effect of arginase inhibition was lost following eNOS inhibition in erythrocytes [3]. Moreover, hearts from eNOS knockout mice were protected when the arginase inhibitor was given with erythrocytes from wild-type donors. In contrast, when hearts from wild-type mice were given with erythrocytes from eNOS knockout mice, the arginase inhibition failed to protect against ischemia-reperfusion [3]. Our findings strongly support the notion that erythrocytes contain functional eNOS and release cardioprotective NO-like bioactivity via a process that is under tight regulation by arginase 1. The eNOS pathway including expression and activity in erythrocytes has been found to be impaired in patients with coronary artery diseases, which significantly correlates with the impaired flow-mediated vasodilation [1]. This suggests that erythrocyte dysfunction with decreased NO bioavailability may affect cardiovascular function. Indeed, our recent findings [4] revealed that erythrocytes from patients and mice with type 2 diabetes induced impaired cardiac tolerance to ischemia-reperfusion. This effect was mediated by increased arginase activity driving eNOS-derived reactive oxygen species (ROS) production by diabetic erythrocytes. Inhibition of erythrocyte arginase in type 2 diabetes not only reduced ROS production by targeting eNOS-uncoupling, but also at high concentrations induced eNOS-dependent protection through increased NO-like bioactivity as in the non-diabetic condition.

Erythrocytes also contribute to the regulation of vascular function by releasing ATP when subjected to hypoxia or shear stress [5]. The release of ATP from erythrocytes requires increases in cAMP, which is hydrolyzed by phosphodiesterase (PDE) 3 [5]. Once released, ATP binds to purinergic receptors on the endothelium to generate vasodilators such as NO and prostacyclin (PGI_2_) [5]. These vasodilators are released extraluminally where they act on vascular smooth muscle to induce vasodilation. NO and PGI_2_ are also released into the vascular lumen where they interact with erythrocytes to inhibit hypoxia-induced ATP release (negative feedback regulation) and stimulate prostacyclin receptor-mediated ATP release (positive feedback regulation), respectively [5]. Notably, the release of ATP from erythrocytes is defective in diseased conditions including diabetes and pulmonary hypertension [5]. This may have implications to affect vascular function. Indeed, erythrocytes from patients with type 2 diabetes fail to dilate resistance vessels under hypoxic conditions in contrast to erythrocytes from healthy subjects [5]. The impairment in vasodilation is restored by PDE3 inhibition in erythrocytes from patients with type 2 diabetes [5], suggesting a detrimental effect of erythrocytes in diabetes on vascular function due to attenuated ATP release. Of further importance, our recent study [6] confirmed a significant role of erythrocytes in the regulation of vascular function in diabetes. We found that erythrocytes from patients with type 2 diabetes were able to induce endothelial dysfunction in both isolated healthy rat aortas and internal mammary arteries from non-diabetic patients undergoing coronary artery bypass grafting. In accordance with our previous findings, endothelial dysfunction was demonstrated *in vivo* in the cohort of patients with type 2 diabetes that donated erythrocytes for the *in vitro* experiments, suggesting that erythrocytes play an important role in the development of endothelial dysfunction observed *in vivo* in type 2 diabetes. In addition to human erythrocytes, erythrocytes from type 2 diabetic rats induced endothelial dysfunction to an extent similar to that observed in the aorta from type 2 diabetic rats. This suggests that it is diabetes *per se* that contributes to the detrimental effect of erythrocytes rather than co-morbidities, co-medication or other confounding factors associated with the group of patients with type 2 diabetes. We further identified arginase 1 and ROS in erythrocytes as key mediators increased endothelial arginase expression and activity resulting in endothelial dysfunction [6]. We have previously shown that arginase inhibition improves both macro- and micro-vascular endothelial function *in vivo* in patients with type 2 diabetes [7,8]. It remains to be determined to what degree inhibition of erythrocyte arginase contributes to this effect.

Our very recent findings confirm and support the previous observations of others that erythrocytes act as active contributors to cardiovascular homeostasis and integrity. These effects are due to the ability of erythrocytes to secrete vasoactive and cardioprotective molecules. Our findings further provide novel knowledge regarding erythrocyte-derived factors, their signaling pathways and how these are altered in diseased conditions for the development of cardiovascular complications. Thus, erythrocyte may serve as a novel therapeutic target in cardiovascular disease.
